# The outcomes of different regimens depend on the molecular subtypes of pulmonary large‐cell neuroendocrine carcinoma: A retrospective study in China

**DOI:** 10.1002/cam4.6834

**Published:** 2024-01-05

**Authors:** Zhaojue Wang, Yang Wu, Tao Lu, Yan Xu, Minjiang Chen, Wei Zhong, Jing Zhao, Mengzhao Wang

**Affiliations:** ^1^ Department of Respiratory and Critical Care Medicine Peking Union Medical College Hospital, Chinese Academy of Medical Sciences and Peking Union Medical College Beijing China; ^2^ School of Medicine Tsinghua University Beijing China; ^3^ Department of Pathology Peking Union Medical College Hospital, Chinese Academy of Medical Sciences and Peking Union Medical College Beijing China

**Keywords:** clinical outcome, molecular subtypes, pulmonary large‐cell neuroendocrine carcinoma, treatment regimen

## Abstract

**Background:**

The optimal systemic treatment for pulmonary large‐cell neuroendocrine carcinoma (LCNEC) remains controversial, and recent advances in LCNEC molecular subtype classification have provided potential strategies for assisting in treatment decisions. Our study aimed to investigate the impact of treatment regimens, molecular subtypes and their concordance on clinical outcomes of patients diagnosed with LCNEC.

**Patients and Methods:**

All patients diagnosed with advanced pulmonary LCNEC in Peking Union Medical College Hospital (PUMCH) between January 2000 and October 2021 were enrolled in this retrospective study. The tumor samples were collected and sequenced using a tumor‐specific gene panel, while clinical information was retrieved from the medical records system. The survival and therapeutic response were analyzed and compared between different subgroups classified by treatment regimen (SCLC or NSCLC‐based), molecular subtype (type I or II) or the combination.

**Results:**

In univariate subgroup analysis categorized only by treatment regimen or molecular subtype, there were no differences identified in DCR, ORR, PFS, or OS. Nevertheless, the group with consistent treatment regimen and molecular subtype exhibited significantly longer OS than that of the inconsistent group (median OS 37.7 vs. 8.3 months; *p* = 0.046). Particularly, the OS of patients with type II LCNEC treated with SCLC‐based regimen was significantly prolonged than that of others (median 37.7 vs. 10.5 months; *p* = 0.039).

**Conclusions:**

Collectively, our study revealed the clinical outcomes of different treatment regimens for LCNEC patients highly depend on their molecular subtypes, highlighting the need for sequencing‐guided therapy.

## 1 INTRODUCTION

Pulmonary large‐cell neuroendocrine carcinoma (LCNEC) is a high‐grade neuroendocrine (NE) tumor and a rare lung cancer subtype with a proportion less than 3%.^1^, ^2^ LCNEC predominantly affects older males with a median age of 65 years and is often associated with a history of heavy smoking.^3^, ^4^ Travis et al. first uncovered LCNEC as a distinct subtype with NE differentiation but different morphological features compared to small cell lung cancer (SCLC).^5^ Subsequent studies have reported that LCNEC is characterized by NE architecture (organoid, nesting, trabeculae, palisade or rosettes), non‐small‐cell cytology (with a cell size three times larger than lymphocytes diameter), low nuclear‐to‐cytoplasmic ratio (abundant cytoplasm and prominent nucleoli), frequent necrosis and high mitotic rate.^6^, ^7^ The histopathological diagnosis of LCNEC relies on the identification of NE morphology and the expression of at least one of the NE markers: neural cell adhesion molecule (NCAM)/CD56, chromogranin A (CgA) and synaptophysin.^8^ NCAM/CD56 is the most sensitive biomarker expressed in 92%–98% of LCNEC cases, but has low specificity due to its expression in other subtypes. In contrast, CgA is the most specific biomarker but with low sensitivity.^9^

The prognosis of patients diagnosed with advanced LCNEC is very poor with a 5‐year survival rate about 10%,^10^ due to not only the highly aggressive property of LCNEC, but also the lack of effective treatment strategies.^11^, ^12^ Given the rarity of LCNEC and its clinicopathological similarities with SCLC and NSCLC, the treatment strategies for LCNEC are primarily based on those applied for SCLC and NSCLC treatment.^4^ The 2015 American Society of Clinical Oncology (ASCO) guidelines provided weak recommendations for platinum plus etoposide or the same treatment for non‐squamous carcinoma as the first‐line treatment for LCNEC.^13^ Since then, the optimal systemic treatment selecting either SCLC or NSCLC based regimens, has still remained a subject of debate, as previous studies focusing on the superiority of the two regimens displayed conflicting results.^7^, ^14^, ^15^, ^16^, ^17^, ^18^

In recent years, the application of next‐generation sequencing in LCNEC tumors has greatly promoted the genomic and transcriptomic characterization of this rare cancer.^19^, ^20^ In general, LCNEC showed a high gene mutation transversion:transition ratio and a high tumor mutation burden. The most pronounced finding was that LCNEC could be categorized into two mutually exclusive groups based on the mutational patterns: type I LCNEC with key genetic alterations of NSCLC (*STK11*/*KEAP1*/*KRAS*) and type II LCNEC with the specific *TP53* and *RB1* co‐mutations of SCLC. Intriguingly, on the transcriptomic level, type I LCNEC shared an SCLC‐like gene expression pattern of ASCL1^high^/DLL3^high^/NOTCH^low^, while type II LCNEC had the opposite feature of ASCL1^low^/DLL3^low^/NOTCH^high^.

The molecular subtype classification reflected the heterogeneity of pulmonary LCNEC, which might lead to complex chemotherapy efficacy reported by previous studies, as different subtypes might predispose the patients to show different therapeutic response.^21^, ^22^ It has been hypothesized that the LCNEC molecular subtypes should be taken into special consideration for the selection of treatment regimens. Thus, in this study, we set out to provide more comprehensive investigation by assessing the impact of treatment regimens, molecular subtypes and their concordance on clinical outcomes in a retrospective cohort of advanced LCNEC patients.

## 2 MATERIALS AND METHODS

### 2.1 Study design and patient selection

This study was conducted in a single‐center, observational, and retrospective cohort, with approval (No. JS‐1410) from the Institutional Review Board (IRB) of Peking Union Medical College Hospital (PUMCH), and informed consent was taken from all the patients. The patients diagnosed with LCNEC or large cell lung cancer (LCLC) in PUMCH from January 2000 to October 2021 were searched in three databases: (1) The Department of Medical Records of PUMCH, (2) the information system of the Department of Pathology of PUMCH, (3) CAPTRA‐LUNG database: a database covering all patients diagnosed with advanced lung cancer in the Department of Respiratory and Critical Care Medicine and Department of Medical Oncology of PUMCH since 2010.

The patients searched above were further selected according to the following inclusion and exclusion criteria. The inclusion criteria were: (1) A confirmed diagnosis of LCNEC: The pathologic diagnosis was reviewed and confirmed according to 2021 the World Health Organization classification of lung tumors,^23^ which included NE morphology (rosettes and peripheral palisading patterns), high mitotic rate (>10 mitoses per 10 high‐power fields), and the expression at least one of the NE markers (CD56, chromogranin A, or Synaptophysin). (2) Stage IIIB or IV according to the eighth edition of the TNM classification for lung cancer of IASLC.^24^ The exclusion criteria were: (1) Combined with other malignant tumors; (2) lack of response evaluation or survival follow‐up. In addition, the baseline liver and renal function results of all enrolled patients were retrieved and identified within the normal range to tolerate chemotherapy toxicities.

### 2.2 Patient characteristics

The patient demographic and clinical characteristics were recorded by reviewing medical records, including: (1) Clinical characteristics: sex, age, smoking/drinking history, previous medical history of chronic disease and cancer, and family history of cancer; (2) Baseline characteristics: symptoms, stage, metastasis site, body mass index (BMI), and ECOG score; (3) Treatment information: treatment regimens, treatment response, and time of disease progression; (4) Survival follow‐up. All patients were followed up until July 2022.

Tumor responses (CR, complete remission; PR, partial response; SD, stable disease; and PD, progressive disease) were evaluated according to the RECIST version 1.1.^12^, ^25^ Disease control rate (DCR) was defined as the percentage of patients whose therapeutic intervention has led to CR, PR, or SD. Objective response rate (ORR) was defined as the percentage of patients with the best overall response of CR or PR relative to the analysis set. Progression‐free survival (PFS) was defined as the duration from diagnosis or assessment before a treatment regimen to the first day of documented disease progression or death. Overall survival (OS) was defined as the time from diagnosis of advanced disease to death of any cause. All 39 patients included in the study had available data for OS. However, due to incomplete data for some patients, the number of patients with available data for PFS, DCR, and ORR was 38, 34, and 32, respectively.

### 2.3 Sample collection, processing and sequencing

Tumor tissue samples were obtained from formalin‐fixed, paraffin‐embedded (FFPE) specimens. DNA was extracted using QIAamp DNA FFPE Tissue Kit (QIAGEN) from 50 to 100 μm of FFPE tumor sections. DNA fragmentation, library preparation, hybridization, and amplification were all performed following the manufacturer's instructions. The DNA was sequenced by Geneplus Seq‐2000 (Geneplus) with the 1021‐Gene Variant Assay. The assay enables variants detection of 1021 genes **(**Table. S1 **)**, which are druggable targets for tumors or are repeatedly reported to be associated with tumor development and progression in the Cancer Genome Atlas (TCGA) & the Catalog of Somatic Mutations in Cancer (COSMIC) database. We performed targeted panel sequencing in 15 samples with sufficient FFPE tissue, among which 12 samples passed quality control and were used for subsequent analysis. The detailed clinical information for all analyzed samples were shown in Table. S2. Specifically, two were obtained from primary tumors through curative surgery, four were obtained from primary tumors through bronchoscopic biopsy, one was obtained from a lymph node from bronchoscopic biopsy, one was obtained from a liver metastatic lesion through CT‐guided needle biopsy, one was obtained from a primary tumor through CT‐guided needle biopsy, two were obtained from cervical lymph nodes through surgical excision, and one was obtained from an abdominal wall nodule through surgical excision. After sequencing, single nucleotide variation (SNV), short insertion and deletion (Indel), copy number variation (CNV), and gene fusions in the target genes were identified for further analysis.

### 2.4 Molecular subtyping

With the sequencing profile, the LCNEC tumors were classified into type I and II according to the following criteria. On the basis of previously reported criteria for type II classification (with both *TP53* and *RB1* co‐mutations),^19^ we incorporated two more SCLC‐unique features (*MYC* family amplification or NOTCH family mutations) according to literature researches and analysis in public datasets. First, both Peifer et al.^26^ and Sos et al.^27^ reported that amplification of MYC family genes were frequent oncogenic events with an alteration frequency of about 20% in SCLC. Second, in the largest available dataset with genomic sequencing data in advanced‐stage tumors, we identified all of *NOTCH1*, *NOTCH2*, and *NOTCH4* mutations were significantly enriched in SCLC compared to NSCLC (Figure. S1). Taken together, the tumor with required TP53 mutation, and any one of the following alterations were classified as type II while others were classified as type I: (1) *RB1* loss‐of‐function mutation, (2) *MYC*/*MYCN* amplification, and (3) *NOTCH* family (*NOTCH1/2/4*) mutations.

### 2.5 Statistical analysis

SPSS software version 27.0 (IBM SPSS Statistics) was used to analyze the results. Categorical variables were shown as numbers and percentages (%). Continuous variables with normal distributions were shown as mean ± standard deviation (SD), and those without normal distributions were shown as median (interquartile range, IQR). Survival curves were generated using the Kaplan–Meier method, and the log‐rank test was used to compare the survival curves. Comparisons were made using the χ2 test, Fisher's test, Student's *t*‐test, Mann–Whitney *U*‐test according to distribution. Statistical significance was defined as a two‐sided *p* < 0.05. The GraphPad Prism software version 9.3 and cBioPortal website^28^, ^29^ (https://www.cbioportal.org) were used for figure generation.

## 3 RESULTS

### 3.1 Establishment of the LCNEC investigation cohort

**FIGURE 1:**
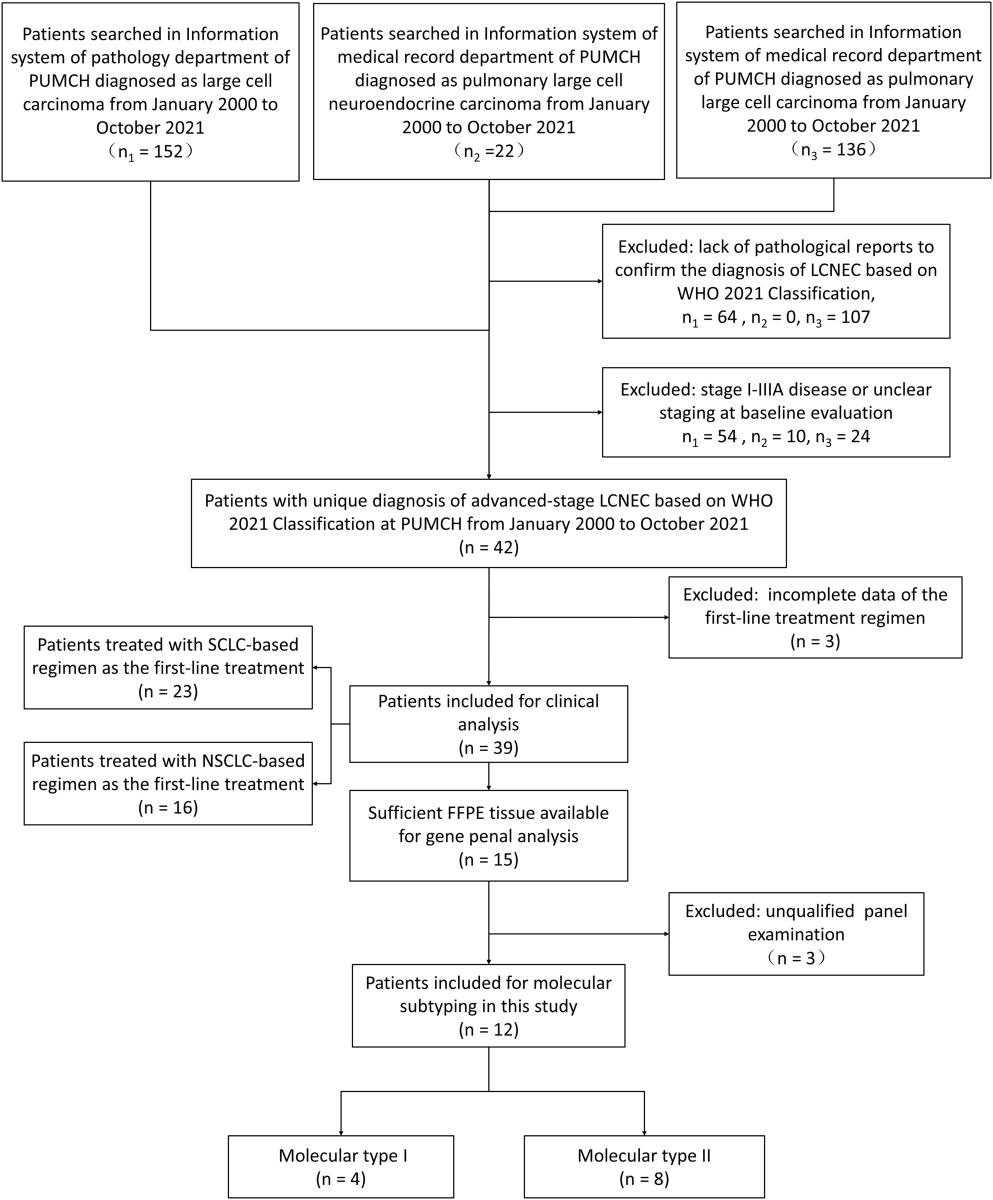
Flow diagram of patient selection and molecular subtyping in this study. The patients diagnosed with advanced pulmonary LCNEC at PUMCH between January 2000 to October 2021 were enrolled in this retrospective study. Clinical analysis and molecular subtyping were performed in entire patients (*n* = 39) and a subgroup of patients (*n* = 12) respectively. LCNEC, large‐cell neuroendocrine carcinoma; NSCLC, non‐small cell lung cancer; PUMCH, Peking Union Medical College Hospital; SCLC, small cell lung cancer.

We established an investigation cohort composed of advanced LCNEC patients receiving systemic therapy through the following procedures (Figure. 1). Briefly, we first searched all patients diagnosed as LCNEC in our center from January 2000 to October 2021, and excluded the patients without pathological report or clear diagnosis as stage IIIB–IV disease. After excluding three patients with incomplete data of first‐line treatment regimen, we finally enrolled a total of 39 patients into this study. The patients were further classified based on the regimens or genetic alterations for subsequent analysis.

The baseline characteristics of LCNEC patients were shown in Table. 1. Notably, 92.3% (36/39) of them were males, 86.8% (33/38) were smokers, 56.4% (22/39) had stage IV disease, and 15.4% (6/39) had recurrent disease after the cured initial stage I/II/II disease. All patients received chemotherapies throughout the entire treatment process. 66.7% (26/39), 28.2% (11/39), and 17.9% (7/39) patients received localized treatment, immunotherapy and targeted therapy respectively.

**TABLE 1:** Baseline characteristics of all LCNEC patients.

	All	SCLC‐based regimen	NSCLC‐based regimen	*p*‐value
Total number	39	23	16	
Gender				0.557
Male, *n* (%)	36 (92.3%)	22/23 (95.7%)	14/16 (87.5%)	
Female, *n* (%)	3 (7.7%)	1/23 (4.3%)	2/16 (12.5%)	
Age (year)	61.3 ± 6.9	60.4 ± 7.4	62.6 ± 6.2	0.328
Background disease, *n* (%)	25/38 (65.8%)	17/23 (73.9%)	8/15 (53.3%)	0.169
Personal history of cancer, *n* (%)	3/38 (7.9%)	2/23 (8.7%)	1/15 (6.7%)	1.000
Family history of cancer, *n* (%)	9/38 (23.7%)	6/23 (26.1%)	3/15 (20.0%)	1.000
Smoking history, *n* (%)	33/38 (86.8%)	21/23 (91.3%)	12/15 (80.0%)	0.365
Symptoms at first visit, *n* (%)	25/39 (64.1%)	14/23(60.9%)	11/16 (68.8%)	0.740
Recurrence, *n* (%)	6/39 (15.4%)	3/23 (13.0%)	3/16 (18.8%)	0.478
Stage				0.743
IIIB–IIIC, *n* (%)	17/39 (43.6%)	11/23 (47.8%)	6/16 (37.5%)	
IVA–IVB, *n* (%)	22/39 (56.4%)	12/23 (52.2%)	10/16 (62.5%)	
BMI (kg/m^2^)	23.44 ± 3.13	23.71 ± 2.90	23.05 ± 3.51	0.552
ECOG score				0.795
0, *n* (%)	19/27 (70.4%)	13/19 (68.4%)	6/8 (75.0%)	
1, *n* (%)	7/27 (25.9%)	5/19 (26.3%)	2/8 (25.0%)	
2, *n* (%)	1/27 (3.7%)	1/19 (5.3%)	0/8 (0.0%)	
Therapy (during follow‐up)				
Immunotherapy, *n* (%)	11/39 (28.2%)	10/23 (43.5%)	1/16 (6.3%)	0.014*
Targeted therapy, *n* (%)	7/39 (17.9%)	2/23 (8.7%)	5/16 (31.3%)	0.101
Localized treatment, *n* (%)	26/39 (66.7%)	16/23 (69.6%)	10/16 (62.5%)	0.736
Therapy (first‐line treatment)				
Immunotherapy, *n* (%)	11/39 (28.2%)	5/23 (21.7%)	1/16 (6.3%)	0.370
Localized treatment, *n* (%)	15/39 (38.5%)	13/23 (56.5%)	2/16 (12.5%)	0.008*

*Note*: Other therapy indicates treatment besides chemotherapy throughout the whole period of follow‐up. Recurrence means the patients experienced recurrence after previous curative surgery and were enrolled at the diagnosis of recurrence. Statistically significant *p*‐values are indicated by asterisks (*p*‐value<0.05).

Abbreviations: BMI, body mass index; NSCLC, non‐small cell lung cancer; SCLC, small cell lung cancer.

**FIGURE 2:**
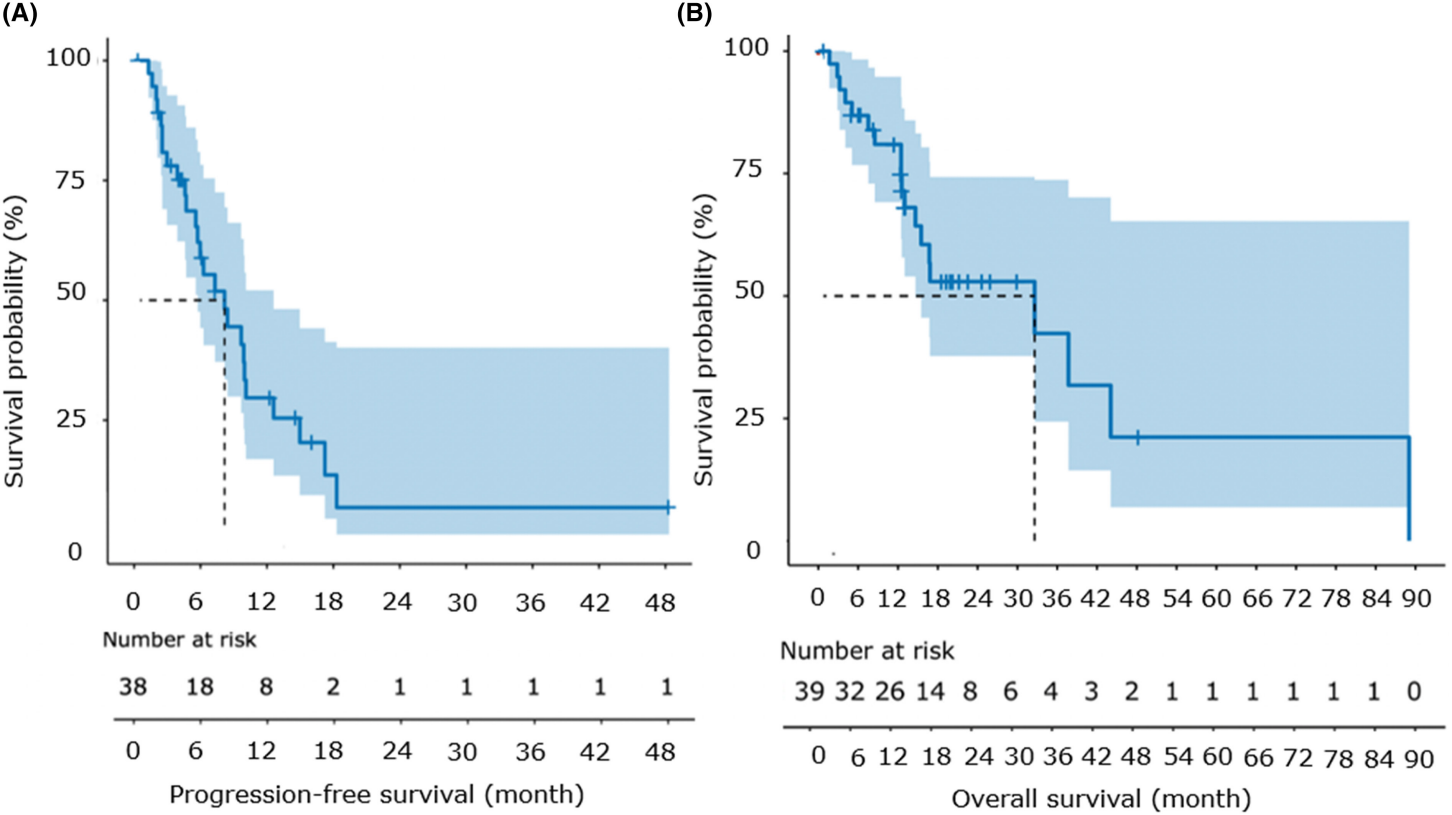
Progression‐free survival and overall survival of the entire cohort. (A, B) Kaplan–Meier plot showing progression‐free survival (PFS) (A) and overall survival (OS) (B) of the entire patients. Thirty eight and 39 patients had available data for PFS and OS respectively. The number of patients at risk was labeled at the bottom of each figure.

At the last follow‐up, 56.4% (22/39) of the patients had succumbed to their illness. For the overall cohort, the progression‐free survival (PFS) and overall survival (OS) curve were shown in Figure. 2, with median PFS (mPFS) of 8.2 months (95% CI: 1.8–11.6 months) and median OS (mOS) of 32.6 months (95% CI: 6.7–58.5 months).

### 3.2 Comparisons between the SCLC‐based regimen and NSCLC‐based regimen

Given that the optimal treatment for LCNEC has not been determined, the first‐line treatment strategies remained highly heterogeneous, so we sought to examine the outcome of different treatments. The patients were divided into two groups according to whether they received SCLC/NSCLC‐based regimen (Figure. 2). Twenty three patients were treated with SCLC‐based regimen, which was defined as etoposide plus platinum or etoposide alone, regardless of combination with immunotherapy or targeted therapy. Sixteen patients were treated with NSCLC‐based regimen, which contained pemetrexed, paclitaxel, docetaxel, gemcitabine or gefitinib. Specifically, for SCLC‐based regimen, 22 patients received etoposide plus platinum (either with carboplatin or cisplatin), and due to intolerance, one patient was treated with etoposide alone. For NSCLC‐based regimen, 6, 4, 2, 2, 1 and 1 patients were treated with pemetrexed plus platinum, paclitaxel plus platinum, docetaxel plus platinum, gefitinib, gemcitabine plus platinum and docetaxel, respectively.

**FIGURE 3:**
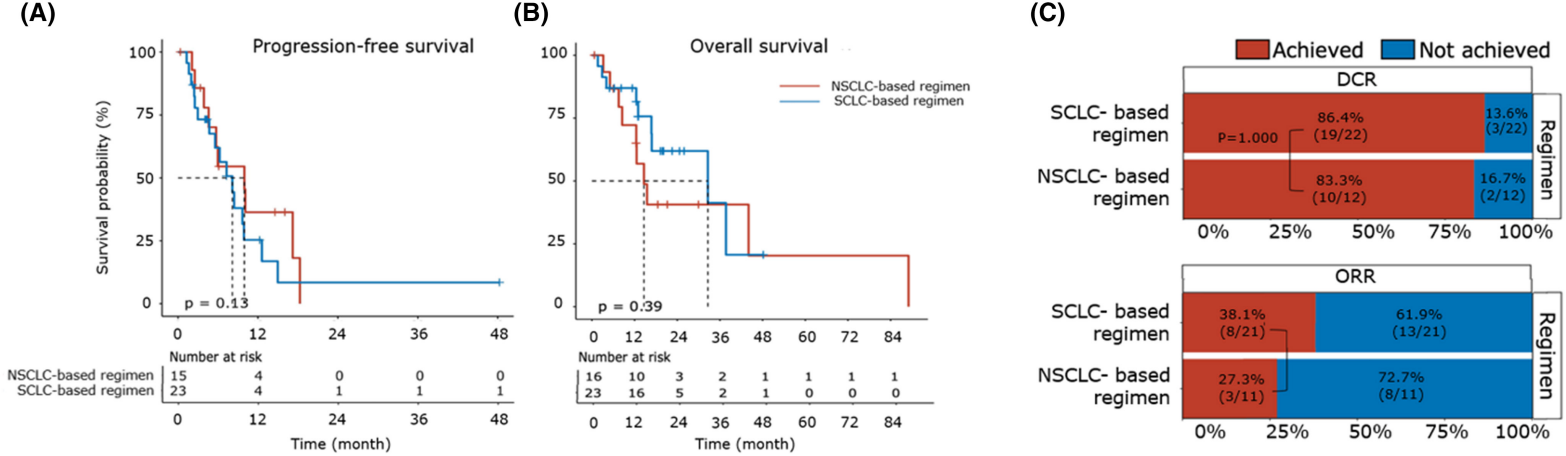
Comparisons between the SCLC‐based regimen and NSCLC‐based regimen. (A, B) Kaplan–Meier plot comparing the progression‐free survival (PFS) (A) and overall survival (OS) (B) between the patients categorized into SCLC‐based regimen and NSCLC‐based regimen groups. Data for PFS (or OS) were available for 23 (or 23) patients in the SCLC‐based regimen group and 15 (or 16) patients in the NSCLC‐based regimen group. The number of patients at risk was labeled at the bottom of each figure. The log‐rank test (two‐sided) was used and the *p*‐value was reported. (C) Stacked bar plot comparing the disease control rate (DCR) (upper part) and objective response rate (ORR) (lower part) between the patients categorized into SCLC‐based regimen and NSCLC‐based regimen groups. Data for DCR (or ORR) were available for 22 (or 21) patients in the SCLC‐based regimen group and 12 (or 11) patients in the NSCLC‐based regimen group. The ratios of patients achieving and not achieving DCR (or ORR) were shown and labeled with red and blue, respectively. Fisher's exact test was used to compare the difference and the *p*‐value was reported. NSCLC, non‐small cell lung cancer; SCLC, small cell lung cancer.

Most baseline clinical characteristics were balanced between the two groups (Table. 1), with the exception that compared to the group treated with NSCLC‐based regimen, there were significantly more (43.5% vs. 6.3%, *p* = 0.014) patients receiving immunotherapy during the follow‐up period, and more patients (56.5% vs. 12.5%, *p* = 0.008) receiving localized treatment (radiotherapy) during the first‐line treatment in the group treated with SCLC‐based regimen. Nonetheless, the clinical outcomes including PFS (median 8.0 vs. 10.0 months; *p* = 0.13), OS (median 32.6 vs. 14.6 months; *p* = 0.39), DCR (86.4% vs. 83.3%; *p* = 1.000) and ORR (38.1% vs. 27.3%; *p* = 0.703) remained similar between these two groups (Figure. 3A–C).

### 3.3 Characterization of the genetic alteration profiles in LCNEC patients

**FIGURE 4:**
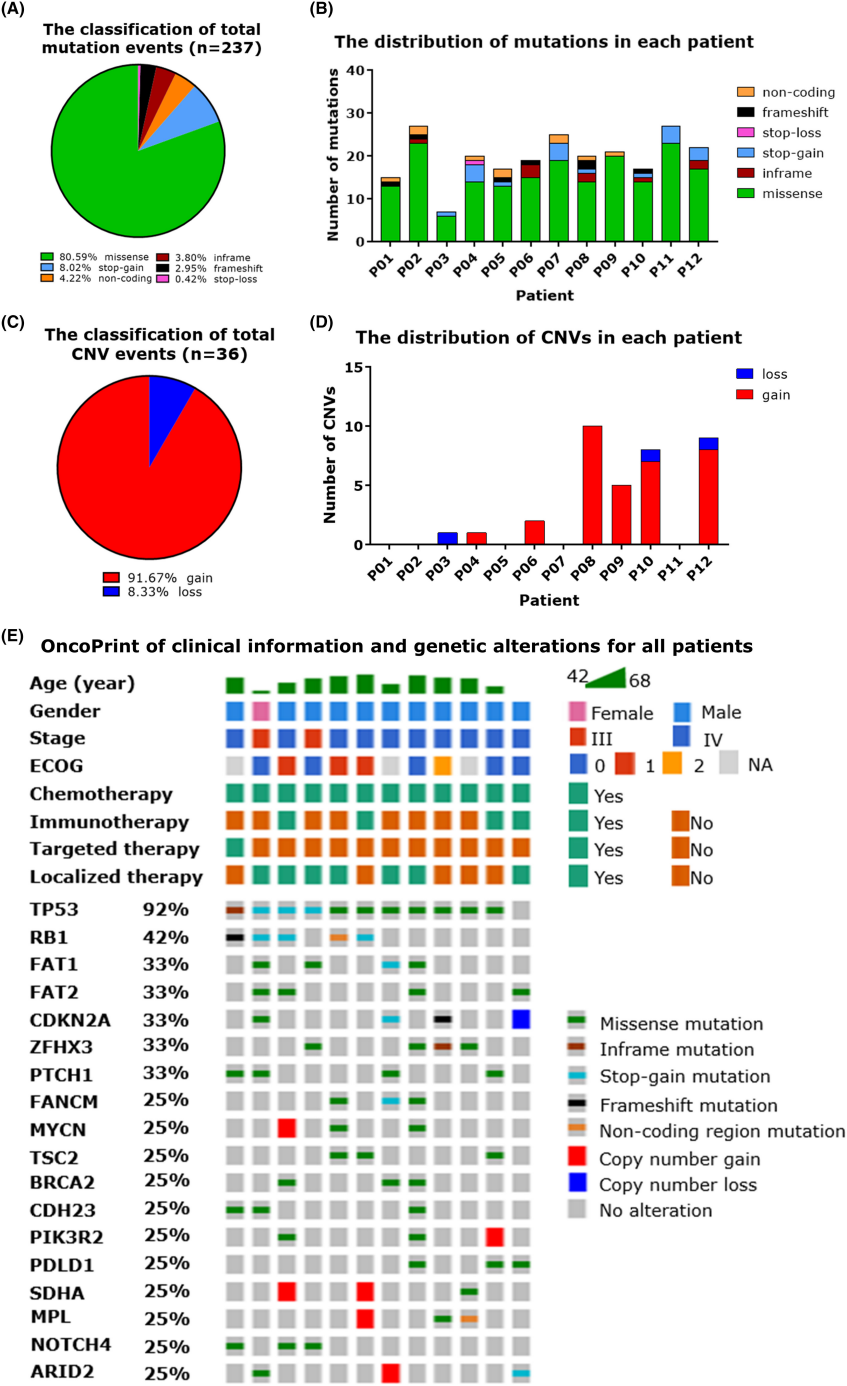
Characterization of the genetic alteration profiles in LCNEC patients. (A) Pie chart showing the composition of all identified mutation events in 12 LCNEC patients. A total of 237 mutation events were classified into six types: missense, inframe, stop‐gain, stop‐loss, frameshift, and non‐coding. The ratio of each type was labeled at the bottom of the figure. (B) Stacked bar plot showing the mutation distribution in each LCNEC patient. The mutation events were classified the same as (A). (C) Pie chart showing the composition of all identified copy number variation (CNV) events in 12 LCNEC patients. A total of 36 CNV events were classified into two types: gain and loss. The ratio of each type was labeled at the bottom of the figure. (D) Stacked bar plot showing the CNV distribution in each LCNEC patient. The CNV events were classified the same as (C). (E) Heatmap displaying the clinical information and genetic alterations for all patients. The clinical characteristics of each patient were depicted on the top, while genetic alterations were highlighted in the corresponding row for genes with alteration frequency less than 25%. LCNEC, large‐cell neuroendocrine carcinoma.

To further characterize the genetic alteration profiles of the LCNEC tumors, we performed targeted panel sequencing and a total of 275 genetic alteration events were identified in 12 samples, including 237 mutations, 36 CNVs and two structural variants. The total mutation events were majorly composed of missense mutations (80.59%), with minor proportion of stop‐gain (8.02%), non‐coding region (4.22%), inframe (3.80%), frameshift (2.95%), and stop‐loss (0.42%) mutations (Figure. 4A). Each patient harbored a certain number of mutations, ranging from 14 to 27 (Figure. 4B). The total CNV events were majorly composed of copy number gains (91.67%), with the minor part of copy number losses (8.33%) (Figure. 4C). Unlike mutations which are carried by every patient, the CNVs were exclusively enriched in seven patients carrying 1 to 10 CNVs (Figure. 4D). Moreover, only two structural variants were detected, including *RET‐KIF5B* fusion in P03 and *ALK‐FSHR* fusion in P05. The top frequent genetic alterations with at least 25% frequency and other clinical information were visualized, showing *TP53* and *RB1* as the top prevalent alterations in all patients (Figure. 4E), and all genetic alterations were provided in Table. S3.

### 3.4 Comparisons between the molecular subtypes of LCNEC

**FIGURE 5:**
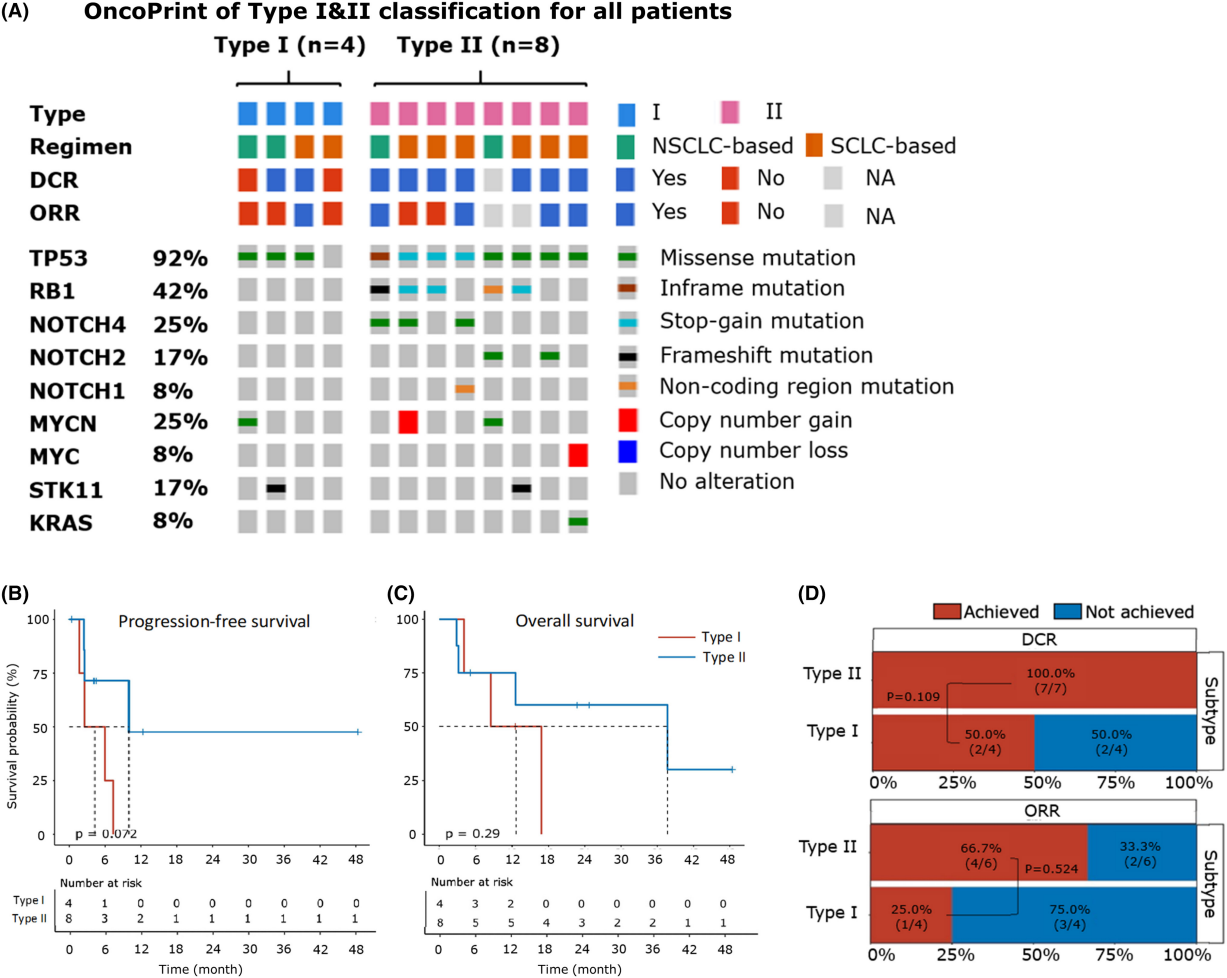
Comparisons between the two molecular subtypes of LCNEC. (A) Heatmap illustrating the molecular subtype classification for four type I and eight type II patients. The treatment regimen and outcome of each patient were depicted on the top, while genetic alterations were highlighted in the corresponding row for criteria genes used for molecular subtyping. (B, C) Kaplan–Meier plot comparing the progression‐free survival (PFS) (B) and overall survival (OS) (C) between type I and type II patients. Data for both PFS and OS were available for four type I and eight type II patients. The number of patients at risk was labeled at the bottom of each figure. The log‐rank test (two‐sided) is used and the *p*‐value was reported. (D) Stacked bar plot comparing the disease control rate (DCR) (upper part) and objective response rate (ORR) (lower part) between type I and type II patients. Data for DCR (or ORR) were available for four (or seven) patients in the consistent group and four (or six) patients in the type II patients. The ratios of patients achieving and not achieving DCR (or ORR) were shown and labeled with red and blue, respectively. Fisher's exact test was used to compare the difference and the *p*‐value was reported.

Previous studies have reported that LCNEC could be divided into two subtypes including type I and type II,^19^, ^21^, ^22^ which had genetic alteration profiles similar to NSCLC and SCLC respectively. In our study, we classified eight samples as type II LCNEC with the required *TP53* mutation, and any one of the following alterations: (1) *RB1* loss‐of‐function mutation, (2) *MYC*/*MYCN* amplification and (3) *NOTCH* family (*NOTCH1/2/4*) mutations (Figure. 5A). Other four samples were classified as type I LCNEC, three of them harbored *STK11* or *KRAS* mutation. For the corresponding first‐line treatment, two type I patients and six type II patients received etoposide with platinum (SCLC‐based regimen), while other two type I patients and one type II patient were treated with pemetrexed‐based chemotherapy (NSCLC‐based regimen), and one type II patient harboring *EGFR* exon19 del received targeted therapy‐gefitinib (NSCLC‐based regimen). The baseline clinical features were all balanced between two subtypes (Table. 2).

**TABLE 2:** Baseline characteristic of the LCNEC patients after molecular profiling.

	All	Type I	Type II	*p*‐value
Total number	12	4	8	
Gender				1.000
Male, *n* (%)	11/12 (91.7%)	4/4 (100.0%)	7/8 (87.5%)	
Female, *n* (%)	1/12 (8.3%)	0/4 (0.0%)	1/8 (12.5%)	
Age (year)	58.91 ± 8.37	56.05 ± 11.03	60.34 ± 7.15	0.430
Background disease, *n* (%)	5/11 (45.5%)	1/4 (25.0%)	4/7 (57.1%)	0.545
Personal history of cancer, *n* (%)	2/11 (18.2%)	0/4 (0.0%)	2/7 (28.6%)	0.491
Family history of cancer, *n* (%)	1/12 (8.3%)	0/4 (0.0%)	0/7 (0.0%)	1.000
Smoking history, *n* (%)	9/11 (81.8%)	4/4 (100.0%)	5/7 (71.4%)	0.491
Symptoms at first visit, *n* (%)	8/12 (66.7%)	2/4 (50.0%)	6/8 (75.0%)	0.547
Recurrence, *n* (%)	3/12 (25.0%)	2/4 (50.0%)	1/8 (12.5%)	0.236
Stage				0.515
IIIB–IIIC, *n* (%)	2/12 (16.7%)	0/4 (0.0%)	2/8 (25.0%)	
IVA–IVB, *n* (%)	10/12 (83.3%)	4/4 (100.0%)	6/8 (75.0%)	
BMI (kg/m^2^)	24.43 ± 3.23	24.93 ± 3.45	24.15 ± 3.34	0.718
ECOG score				0.165
0, *n* (%)	5/9 (55.6%)	3/3 (100.0%)	2/6 (33.3%)	
1, *n* (%)	3/9 (33.3%)	0/3 (0.0%)	3/6 (50.0%)	
2, *n* (%)	1/9 (11.1%)	0/3 (0.0%)	1/6 (16.7%)	
Therapy (during follow‐up)				
Immunotherapy, *n* (%)	4/12 (33.3%)	2/4 (50.0%)	2/8 (25.0%)	0.547
Targeted therapy, *n* (%)	1/12 (8.3%)	0/4 (0.0%)	1/8 (12.5%)	1.000
Localized treatment, *n* (%)	7/12 (58.3%)	2/4 (50.0%)	5/8 (62.5%)	1.000
Therapy (first‐line treatment)				
Chemotherapy, *n* (%)	9/12 (75.0%)	3/4 (75.0%)	6/8 (75.0%)	1.000
Chemotherapy with immunotherapy, *n* (%)	2/12 (16.7%)	1/4 (25.0%)	1/8 (12.5%)	1.000
Gefitinib, *n* (%)	1/12 (8.3%)	0/4 (0.0%)	1/8 (12.5%)	1.000
Localized treatment, *n* (%)	2/12 (16.7%)	0/4 (0.0%)	2/8 (25.0%)	0.515

*Note*: Recurrence means the patients experienced recurrence after previous curative surgery and were enrolled at the diagnosis of recurrence. Here the one case with targeted therapy referred to the EGFR inhibitor, gefitinib. Statistically significant *p*‐values are indicated by asterisks (*p*‐value<0.05).

Abbreviations: BMI, body mass index; NSCLC, non‐small cell lung cancer; SCLC, small cell lung cancer.

Patients with type II LCNEC tended to have longer PFS (median 10.0 vs. 5.0 months; *p* = 0.072) than patients with type I LCNEC (Figure. 5B). There was no significant difference on the DCR (100.0% vs. 50.0%; *p* = 1.000), ORR (66.7% vs. 25.0%; *p* = 0.524), and OS (median 37.7 vs. 14.7 months; *p* = 0.294) between two groups (Figure. 5C,D).

### 3.5 Integrative prognostic analysis of molecular subtype‐treatment regimen concordance

**FIGURE 6:**
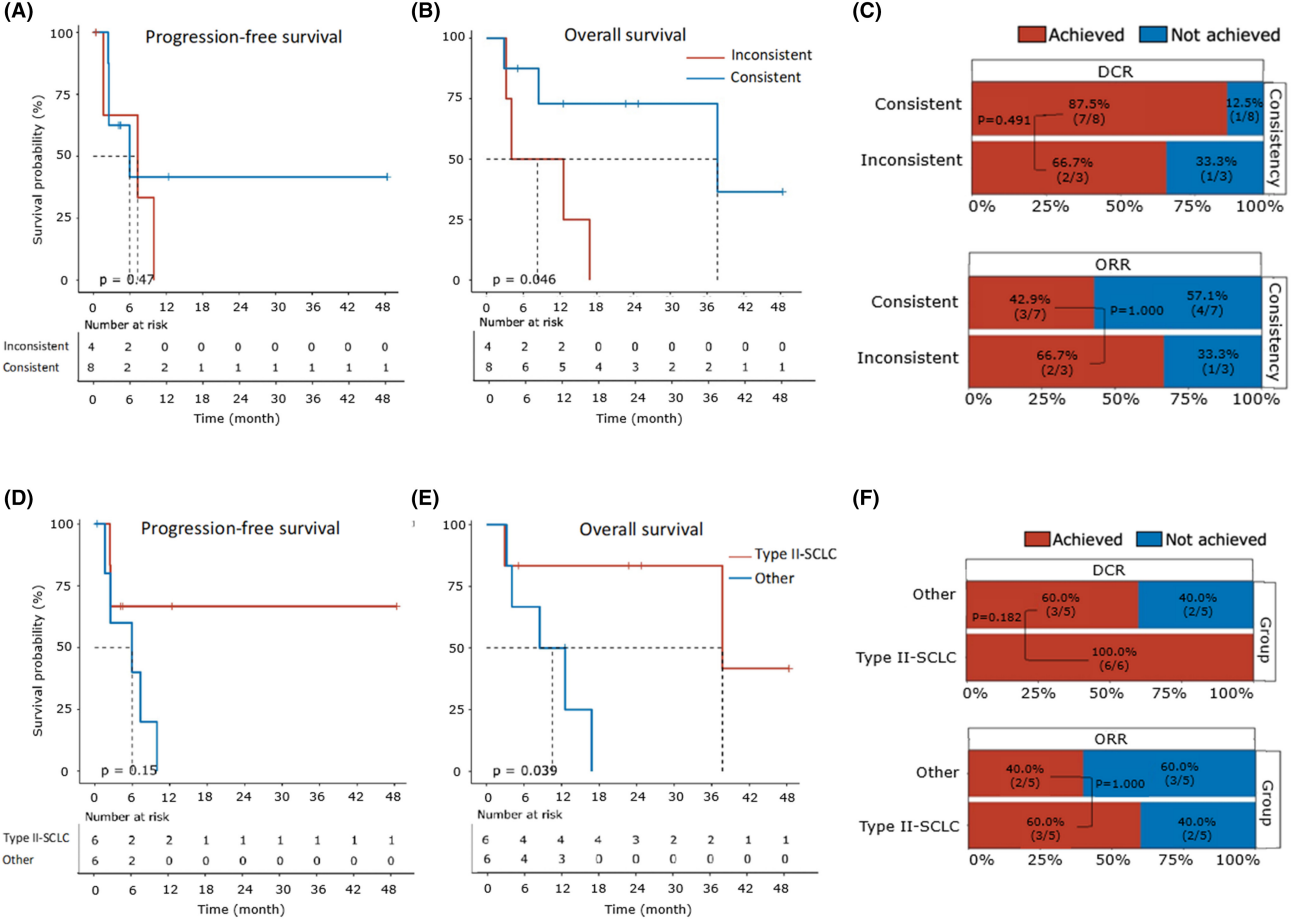
Integrative prognostic analysis of the molecular subtype‐treatment regimen concordance. (A, B) Kaplan–Meier plot comparing the progression‐free survival (PFS) (A) and overall survival (OS) (B) between the patients categorized into consistent and inconsistent groups. Data for both PFS and OS were available for eight patients in the consistent group and four patients in the inconsistent group. The number of patients at risk is labeled at the bottom of each figure. The log‐rank test (two‐sided) was used and the *p*‐value was reported. (C) Stacked bar plot comparing the disease control rate (DCR) (upper part) and objective response rate (ORR) (lower part) between the patients categorized into consistent and inconsistent groups. Data for DCR (or ORR) were available for eight (or seven) patients in the consistent group and three (or three) patients in the inconsistent group. The ratios of patients achieving and not achieving DCR (or ORR) were shown and labeled with red and blue, respectively. The Fisher's exact test was used to compare the difference and the p‐value was reported. (D, E) Kaplan–Meier plot comparing the progression‐free survival (PFS) (D) and overall survival (OS) (E) between the type II patients treated with SCLC‐based regimen (type II‐SCLC) and other patients. Data for both PFS and OS were available for six patients in the type II‐SCLC group and six patients in the other group. The number of patients at risk was labeled at the bottom of each figure. The log‐rank test (two‐sided) was used and the *p*‐value was reported. (F) Stacked bar plot comparing the DCR (upper part) and ORR (lower part) between the type II patients treated with SCLC‐based regimen (type II‐SCLC) and other patients. Data for DCR (or ORR) were available for six (or five) patients in the type II‐SCLC group and five (or five) patients in the other group. The ratios of patients achieving and not achieving DCR (or ORR) were shown and labeled with red and blue, respectively. Fisher's exact test was used to compare the difference and the *p*‐value was reported.

To comprehensively analyze the impact of the correspondence between molecular subtype and treatment regimen on therapeutic outcome, we first divided the 12 patients into the consistent group (type I LCENC treated with NSCLC‐based regimen or type II LCNEC treated with SCLC‐based regimen) and the inconsistent group (type I LCNEC treated with SCLC‐based regimen or type II LCNEC treated with NSCLC‐like regimen). The two groups included eight patients and four patients respectively, and their baseline characteristics were balanced (Table. 3). The OS of the consistent group was significantly longer than that of the inconsistent group (median 37.7 vs. 8.3 months; *p* = 0.046), with no significant difference on the PFS (median 7.8 vs. 7.4 months; *p* = 0.468), DCR (87.5% vs. 66.7%; *p* = 0.491) and ORR (42.9% vs. 66.7%; *p* = 1.000) (Figure. 6A–C).

**TABLE 3:** Baseline characteristic of LCNEC patients classified by molecular subtype‐treatment regimen concordance.

	Inconsistent	Consistent	*p*‐value	Type II‐SCLC	Other	*p*‐value
Total number	4	8		6	6	
Gender			1.000			1.000
Male, *n* (%)	4/4 (100.0%)	7/8 (87.5%)		5/6 (83.3%)	6/6 (100.0%)	
Female, *n* (%)	0/4 (0.0%)	1/8 (12.5%)		1/6 (16.7%)	0/6 (0.0%)	
Age(year)	56.05 ± 11.03	60.34 ± 7.15	0.430	58.83 ± 7.74	58.99 ± 9.71	0.977
Background disease, *n* (%)	2/3 (66.7%)	3/8 (37.5%)	0.545	3/6 (50.0%)	2/5 (40.0%)	1.000
Personal history of cancer, *n* (%)	1/3 (33.3%)	1/8 (12.5%)	0.491	1/6 (16.7%)	1/5 (20.0%)	1.000
Family history of cancer, *n* (%)	0/3 (0.0%)	0/8 (0.0%)	1.000	0/6 (0.0%)	0/5 (0.0%)	1.000
Smoking history, *n* (%)	3/3 (100.0%)	6/8 (75.0%)	1.000	4/6 (66.7%)	5/5 (100.0%)	0.491
Symptoms at first visit, *n* (%)	3/4 (75.0%)	5/8 (62.5%)	1.000	4/6 (66.7%)	4/6 (66.7%)	1.000
Recurrence, *n* (%)	1/4 (25.0%)	2/8 (25.0%)	1.000	1/6 (16.7%)	2/6 (33.3%)	1.000
Stage			0.515			0.455
IIIB–IIIC, *n* (%)	0/4 (0.0%)	2/8 (25.0%)		2/6 (33.3%)	0/6 (0.0%)	
IVA–IVB, *n* (%)	4/4 (100.0%)	6/8 (75.0%)		4/6 (66.7%)	6/6 (100.0%)	
BMI(kg/m^2^)	24.93 ± 3.45	24.15 ± 3.34	0.718	24.14 ± 3.66	24.79 ± 3.01	0.483
ECOG score			0.741			0.487
0, *n* (%)	2/3 (66.7%)	3/6 (50.0%)		2/6 (33.3%)	3/6 (50.0%)	
1, *n* (%)	1/3 (33.3%)	2/6 (33/3%)		2/6 (33.3%)	1/6 (0.0%)	
2, *n* (%)	0/3 (0.0%)	1/6 (16.7%)		1/6 (16.7%)	0/6 (0.0%)	
Therapy (during follow‐up)						
Immunotherapy, *n* (%)	2/4 (50.0%)	2/8 (25.0%)	0.547	2/6 (33.3%)	2/6 (33.3%)	1.000
Targeted therapy, *n* (%)	1/4 (25.0%)	0/8 (0.0%)	0.333	0/6 (0.0%)	1/6 (0.0%)	1.000
Localized treatment, *n* (%)	2/4 (50.0%)	5/8 (62.5%)	1.000	4/6 (66.7%)	3/6 (50.0%)	1.000
Therapy (first‐line treatment)						
Chemotherapy, *n* (%)	2/4 (50.0%)	7/8 (87.5%)	0.236	5/6 (83.3%)	4/6 (66.7%)	1.000
Chemotherapy with immunotherapy, *n* (%)	1/4 (25.0%)	1/8 (12.5%)	1.000	1/6 (16.7%)	1/6 (0.0%)	1.000
Gefitinib, *n* (%)	1/4 (25.0%)	0/8 (0.0%)	0.333	0/6 (0.0%)	1/6 (0.0%)	1.000
Localized treatment, *n* (%)	0/4 (0.0%)	2/8 (25.0%)	0.515	2/6 (33.3%)	0/6 (0.0%)	0.455

*Note*: Recurrence means the patients experienced recurrence after previous curative surgery and were enrolled at the diagnosis of recurrence. Here the one case with targeted therapy referred to the EGFR inhibitor, gefitinib. Statistically significant p values are indicated by asterisks (*p*‐value<0.05).

Abbreviations: BMI, body mass index; NSCLC, non‐small cell lung cancer; SCLC, small cell lung cancer.

We then specially focused on the group composed of type II patients treated with SCLC‐based regimen (type II‐SCLC), and compared its prognosis with others (all type I patients and type II patients treated with NSCLC‐based regimen). Their baseline characteristics were also shown in Table. 3. Notably, the type II patients treated with SCLC‐like regimen exhibited significantly longer OS than that of others (median 37.7 vs. 10.5 months, *p* = 0.039), while the DCR (87.5% vs. 100.0%; *p* = 0.182), ORR (40.0% vs. 60.0%; *p* = 1.000), and PFS (median 7.8 vs. 7.4 months, *p* = 0.154) remained similar (Figure. 6D–F). Univariate analyses were also performed for other variables, and no other factors exhibited a significant association with PFS or OS (Table. S4).

## 4 DISCUSSION

Here we explored the optimal therapeutic strategy for advanced LCNEC in a retrospective cohort of 39 patients, and further profiled the genetic alterations in a group of 12 patients. The patients were classified into subgroups based on molecular subtypes (type I vs. type II) or treatment regimens (NSCLC‐based regimen vs. SCLC‐based regimen). Our integrative analysis revealed the concordance of molecular subtypes and treatment regimens could significantly prolong the survival of patients, with the potential to guide precise treatment.

We have modified the criteria for type II LCNEC classification, which not only depended on the alteration status of the *TP53* and *RB1* genes, but also included two molecular features commonly reported in SCLC rather than NSCLC: *MYC* family amplifications and *NOTCH* family mutations. Since, Rekhtman et al. first reported the two molecular subtypes of LCNEC distinguished by the *TP53* and *RB1* co‐mutations,^19^ then subsequent clinical researches have followed their criteria for LCNEC classification. Given more complicated genomic and transcriptomic landscapes were profiled for LCNEC,^20^ its intermediate features have posed a challenge to the previous classification criteria simply based on *TP53* and *RB1* co‐mutations. We made the modification based on the observation that some patients, classified as type I LCNEC according to the previous criteria, also showed favorable responses to SCLC‐based chemotherapy. Remarkably, these patients also exhibited characteristic mutations of SCLC, aside from the co‐mutation of RB1 and TP53. In our study, 3/12 (25%) samples harbored no *TP53* and *RB1* co‐mutations, but *MYC* family amplifications or *NOTCH* family mutations. They were classified as type II LCNEC according to our modified criteria, and treated with consistent SCLC‐based chemotherapy. Thus we speculated that our criteria might increase the sensitivity to classify all the LCNEC patients with promising response to SCLC‐based regimen as the type II group. Moreover, the current understanding of molecular subtyping for LCNEC is still ongoing, it is necessary to examine and improve the criteria in large or real‐world studies.

Our study has highlighted the importance of classifying patients through molecular subtyping to evaluate their responses to different treatment regimens. Initially, among the overall 39 LCNEC patients without molecular subtyping, neither SCLC nor NSCLC‐based regimens exhibited superior therapeutic efficacy. Nevertheless, by incorporating genetic alteration profiles from 12 patients, we could perform more comprehensive comparisons by integrating the molecular subtypes and treatment regimens, and observed significantly prolonged OS in the consistent group (vs. the inconsistent group), particularly in the type II treated with SCLC‐based regimen group (vs. others). No difference in OS was observed between type I and type II patients, thus we hypothesize that instead of regarding molecular subtype or treatment regimen as an individual prognostic factor, the concordance of them was a promising indicator for more favorable clinical outcome.

Furthermore, the concordance of molecular subtype and treatment regimen could be specifically divided into two conditions: NSCLC‐like LCNEC treated with NSCLC‐based regimen and SCLC‐like LCNEC treated with SCLC‐based regimen. The prognosis of the two conditions should be investigated separately, and previous studies have reported some results for our reference. Zhou et al. revealed that compared to pemetrexed plus platinum or gemcitabine/taxane plus platinum (NSCLC‐based regimens), etoposide plus platinum (SCLC‐based regimen) was associated with superior response and survival in patients with SCLC‐like LCNEC. Intriguingly, in patients with NSCLC‐like LCNEC, etoposide plus platinum still worked well while gemcitabine/taxane plus platinum led to a shorter survival.^30^ The second finding was contradictory to the study by Derks et al. which observed patients bearing LCNEC tumors with wild‐type RB1 gene or expression of RB1 protein achieved longer survival when treated with gemcitabine/taxane plus platinum than those treated with platinum and etoposide.^31^ The results of our study in part supported Zhuo et al. that we consistently identified better clinical outcome in the SCLC‐like LCNEC group treated with SCLC‐based regimen. For the controversial part about NSCLC‐like LCNEC, we did not provide additional strong evidence as only four patients were classified as this subtype in our study. Indeed, the previous two studies both had small sample size of NSCLC‐like LCNEC due to its relative rareness, so such discrepancy should be interpreted carefully and future studies with large sample size are required to address this issue.

Another interesting finding is the favorable outcomes that the patients with type II LCNEC in our cohort had the mPFS of 10.0 months and mOS of 37.7 months, which surpassed most of previous studies showing mPFS ranged from 4.4 to 6.1 months and mOS ranged from 8.0 to 51 months in patients with advanced LCNEC.^14^, ^32^, ^33^, ^34^, ^35^ We speculated the reasons could be: (1) A large portion of patients were treated with consistent SCLC‐based regiment, aligning with the study by Rossi et al.^32^ which reported the highest mOS of 51 months in patients treated with SCLC‐based chemotherapy too. (2) Some patients underwent systematic therapy, including immunotherapy and localized therapy, in accordance with recent researches indicating their potential benefits in improving the survival of LCNEC patients.^36^, ^37^, ^38^ Thus both of the two factors could potentially contribute to longer PFS/OS in our study. Notably, there were great difference in survival of LCNEC patients across different studies, suggesting the limited sample size and variations in patient characteristics could cause bias to the results.

In our study, the potential bias caused by other factors could not be excluded, it is not possible to control for all variables due to the limited sample size. Given the rarity of LCNEC, it is extremely challenging to collect a sufficient size of LCNEC samples in a single‐center study. Our study retrieved clinical data of 39 LCNEC patients, which is similar to the sample size in previous LCNEC studies. Moreover, we performed comprehensive molecular characterization for 12 patients, revealing the association between subtype‐regimen consistency and survival. While the confidence of our conclusion might be impacted by other biases, our study provides preliminary findings and novel insights into such an understudied but controversial area. Collectively, our study was limited by the single‐center source and relatively small sample size, emphasizing the need for future validation through larger‐cohort or multi‐center studies. In addition, it was a retrospective study spanning a considerable timeframe, thus high‐quality prospective studies are warranted to mitigate the impact of other confounding factors like treatment advancements.

## 5 CONCLUSION

To conclude, the clinical outcomes of different treatment regimens for LCNEC patients highly depend on their molecular subtypes. The consistency of the two factors would bring remarkable survival advantages to LCNEC patients, especially for type II LCNEC patients treated with SCLC‐based regimen. Taken together, our study provided novel insights for the LCNEC precise treatment, pressing the need for genetic characterization to guide further therapy.

## AUTHOR CONTRIBUTIONS

**Zhaojue Wang:** Conceptualization (equal); data curation (equal); formal analysis (equal); resources (equal); visualization (equal); writing – original draft (equal). **Yang Wu:** Data curation (equal); formal analysis (equal); visualization (equal); writing – original draft (equal); writing – review and editing (equal). **Tao Lu:** Resources (supporting); writing – review and editing (supporting). **Yan Xu:** Resources (supporting); writing – review and editing (supporting). **minjiang chen:** Resources (supporting); writing – review and editing (supporting). **Wei Zhong:** Resources (supporting); writing – review and editing (supporting). **Jing Zhao:** Conceptualization (equal); funding acquisition (equal); project administration (equal); resources (equal); supervision (equal); writing – review and editing (equal). **Mengzhao Wang:** Conceptualization (equal); funding acquisition (equal); resources (equal); supervision (equal); writing – review and editing (equal).

## FUNDING INFORMATION

This work was supported by National High Level Hospital Clinical Research Funding (No. 2022‐PUMCH‐A‐011, to Jing Zhao) and the CAPTRA‐Lung Research Funds (No. CAPTRALung2021005, to Jing Zhao). The authors declare that they have no known competing financial interests or personal relationships that could have appeared to influence the work reported in this paper.

## Supporting information


Figure S1.
Figure S2.

Table S1.

Table S2.

Table S3.

Table S4.

## Data Availability

The data that support the findings of our study are included within the article, other raw data are available from the corresponding author upon reasonable request.
