# Supplementary material for: The outcomes of different regimens depend on the molecular subtypes of pulmonary large‐cell neuroendocrine carcinoma: A retrospective study in China
Source: Cancer Med. 2024 Jan 5;13(1):e6834. doi: 10.1002/cam4.6834 (PMC10807557; doi:10.1002/cam4.6834)
Supplement: Supplementary file 1 — Figure S1. Figure S2. [file CAM4-13-e6834-s005.pptx]

## Slide 1
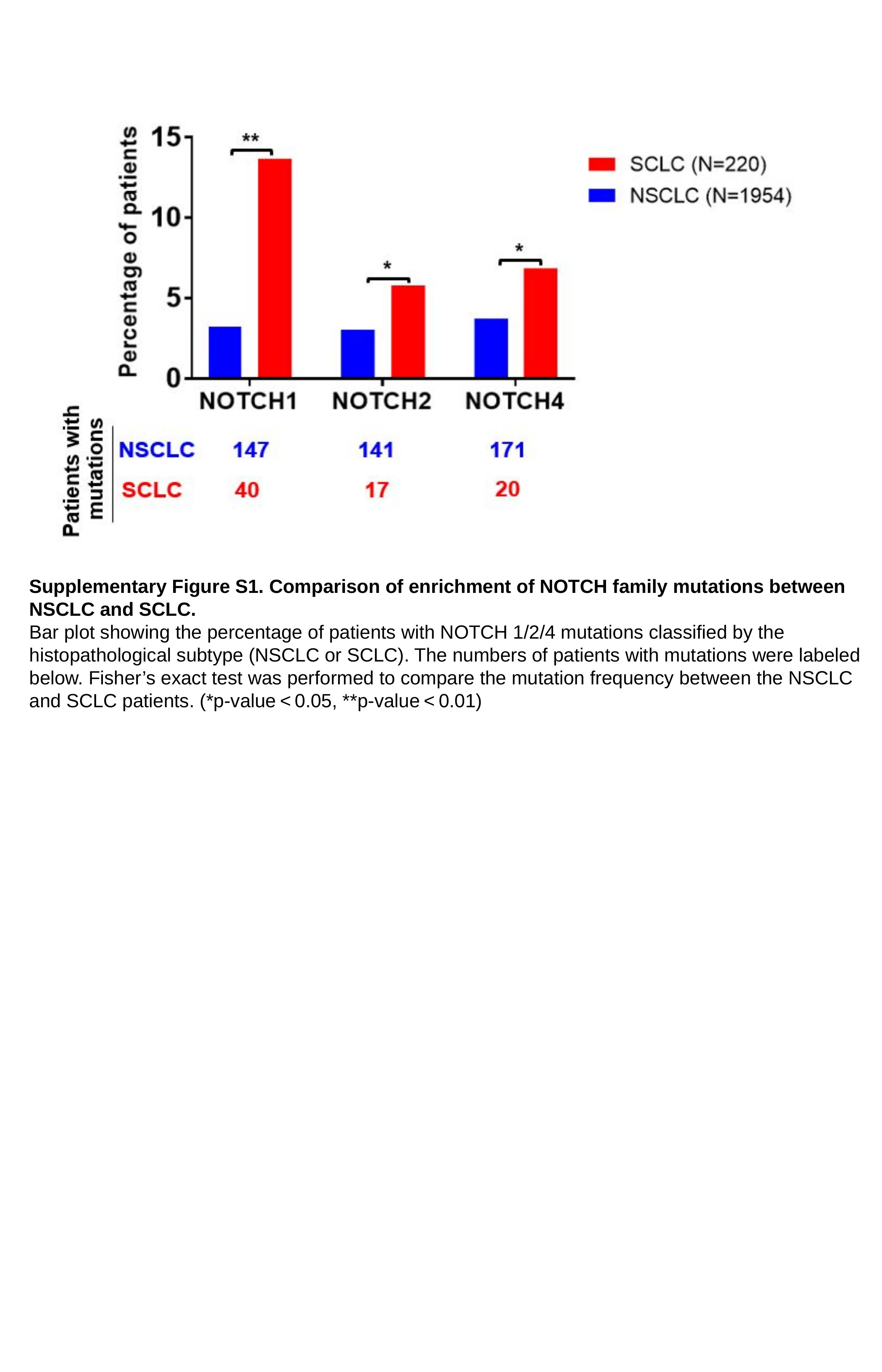

Supplementary Figure S1. Comparison of enrichment of NOTCH family mutations between NSCLC and SCLC.
Bar plot showing the percentage of patients with NOTCH 1/2/4 mutations classified by the histopathological subtype (NSCLC or SCLC). The numbers of patients with mutations were labeled below. Fisher’s exact test was performed to compare the mutation frequency between the NSCLC and SCLC patients. (*p-value < 0.05, **p-value < 0.01)

## Slide 2
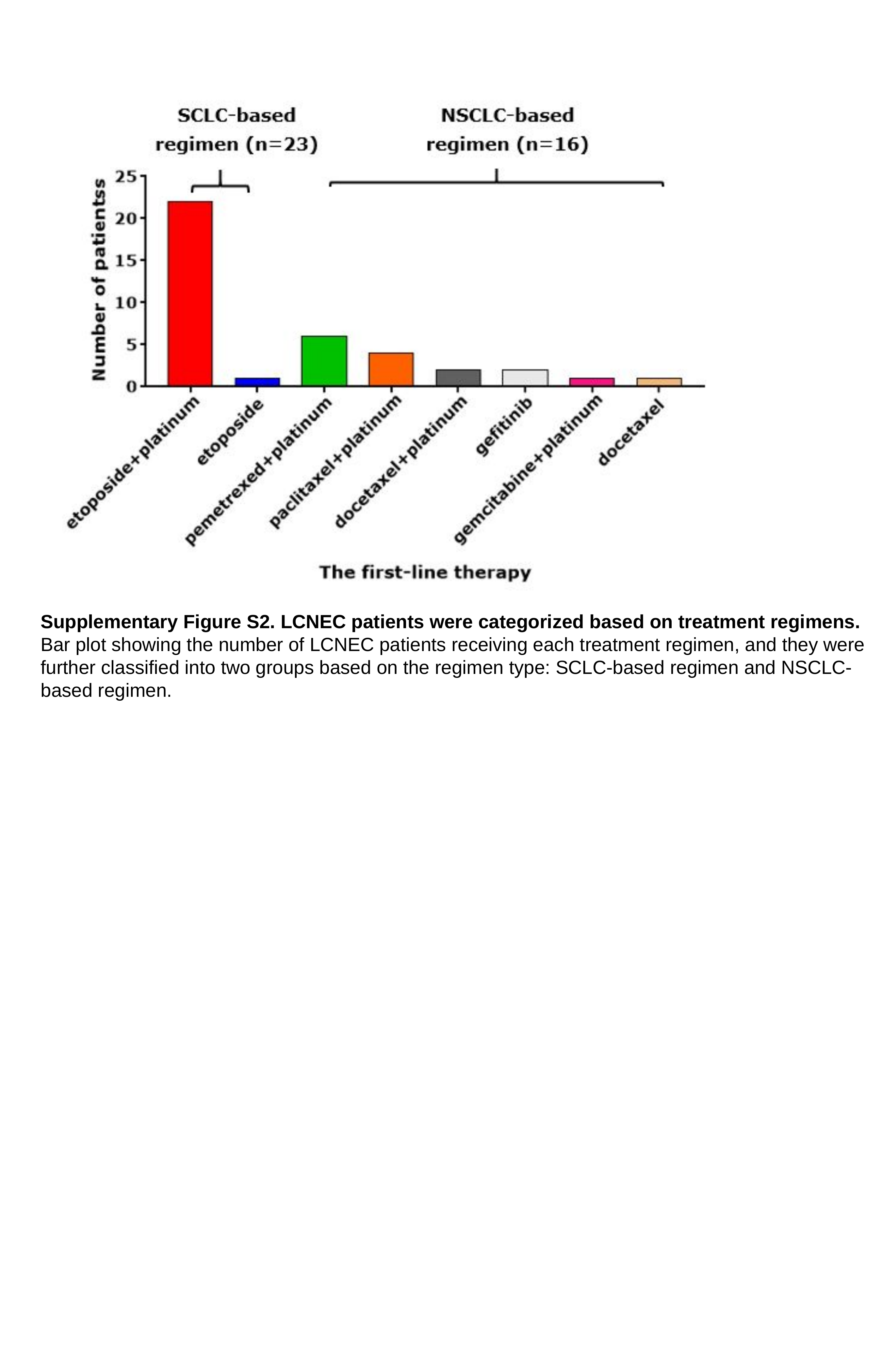

Supplementary Figure S2. LCNEC patients were categorized based on treatment regimens.
Bar plot showing the number of LCNEC patients receiving each treatment regimen, and they were further classified into two groups based on the regimen type: SCLC-based regimen and NSCLC-based regimen.
